# Automatic Arrangement of Sports Dance Movement Based on Deep Learning

**DOI:** 10.1155/2022/9722558

**Published:** 2022-02-10

**Authors:** Hua Feng, Xiang Zhao, Xiaomin Zhang

**Affiliations:** ^1^College of Physical Education, Huaibei Normal University, Huaibei 235000, Anhui, China; ^2^Graduate School, Adamson University, Manila 0900, Philippines; ^3^Electronics and Information Engineering, Huaibei Institute of Technology University, Huaibei 235000, Anhui, China

## Abstract

Sports dance is a new form of sports that integrates sports, dance, music, and other elements. The core content of “dance” is an important carrier for athletes to display their body art. This article aims to study the automatic arrangement of sports dance based on deep learning. This article first introduces the development process of deep learning. As the latest research direction developed from artificial neural network technology in machine learning, deep learning has attracted widespread attention from the society. And then proposing a shallow regression model based on deep learning, a convolutional neural network based on deep learning, and an offline sorting regression model, given the general process of deep learning, then, based on the clustering algorithm, the deep learning was researched, and the sport dance movement arrangement was analyzed based on the deep learning. The experimental results of this article show that deep learning can effectively enhance the artistic ability of automatic choreography in sports dance and increase the accuracy of dance movements by 80%. At the same time, on the basis of deep learning, the practical ability is strengthened on the basis of consolidating theory, to further improve one's own business ability and educational technology level, actively absorb advanced teaching methods, and earnestly delve into reasonable teaching methods. It is also used in curriculum training practice to actively gain insight into new development trends in educational methods and skills, to enhance the artistic creativity of students' arrangements.

## 1. Introduction

Sports dance is the most innovative way of sports in the field of sports in the world today. Its elegant dance steps, special clothing decorations, and cheerful song rhythm have aroused strong interest among people all over the world. At the same time, it also sparked a wave of learning sports and dance. With the in-depth discussion and development of sports dance theory in the world, the aesthetic standard of sport has gradually returned from the improvement of movement skills to the aesthetic essence of dance movements. It not only emphasizes the training and improvement of the athletes' physical fitness, but also begins to pay more attention to the expression of their emotions and ways of thinking in the creation of sports dance and pays more attention to the infiltration of the athletes' own artistic expression and inner emotions in the dance movements. Sports dance is also a typical sports event that combines “health” and “beauty.” It is loved by the masses for its innovative activity forms, rich cultural content, and superartistic appeal. For example, Guangzhou and Shanghai, which were the first places to launch sports art dances, are under the background of the national fitness spirit, and there has been a big wave of practicing sports art dances for people, and outstanding professional players are also emerging in an endless stream. Sports art dance has gradually become one of the competitive sports events of sports. At the same time, as the formation of Olympic performance events and Asian Games competition events, it has also received the attention of the State Sports General Administration. In more aspects, the support and guarantee of national policies have been increased, which has greatly promoted the vigorous development of sports, art, and dance. How does sports dance show such a beautiful artistic form through the body? This needs to be studied from the root of its physical movement characteristics. Thus, finding the law of physical movement characteristics can not only understand the root cause of the beauty of sports dance, but also find the corresponding training methods to produce the beauty of sports dance more appropriately.

Sports art performance projects are the unity of internal spiritual temperament and external dynamic expression and are the highest level of performance in performance projects. Dance is a performance art form with body movement as the main performance method, and beauty is an essential feature of dance aesthetics. Through deep learning, this article has conducted an in-depth research on the automatic arrangement of sports dance sports and provided theoretical support for cultivating sports dance players' competitive talents and developing sports dance careers. At the same time, the development of sports dance provides people with opportunities to study physical and mental development and interpret artistic creativity, costume design, and dance design through music.

According to the research progress at home and abroad, different scholars have also made corresponding investigations in deep learning and automatic arrangement of sports dances: after analysis, Jing H pointed out that the continuous dynamics of two independent motion streams constitute a shared dancing experience. In this sense, exercising together in a sports and dance is a practical way to get to know each other. It also emphasizes the two-way nature of sharing and reveals how to deliberately shape reciprocity through mutual coordination and emotional restraint of movement [1]. Poutanen describes a user-centered design method that extends the idea of orchestration to interaction design and shows how to perform micromotion analysis in practice. We use the structural reorganization of the continuum of motion that originally appeared in the video to perform the arrangement in the first person as a means of understanding the kinesthetic quality and implicit arrangement potential. This method emphasizes the influence of interaction design on mobile and experiencing bodies, and the potential of mobile and experiencing bodies on interaction design [2]. Liu introduced the basic connotations of the ant colony theory, at the same time, analyzed the development status of modern music and dance technique movement matching optimization, and further proposed a simulation model of modern music and dance technique movement. It aims to improve the matching degree of modern music and dance movement skills and promote the development of modern choreography from the aspects of implementing information collection and constructing modern music and dance technical action matching optimization process [3]. The purpose of the Jarvis study is to review the relevant literature and describe how the movement patterns of trained dancers are affected by induced acute physical fatigue. Research on dancer fatigue found that there are conflicting results in the transfer contribution of lower limb joints, which indicates that more research is needed to determine the effect of fatigue on dancers' jumping mechanics [4]. Shen proposed a 50 fps real-time image superpixel segmentation method by using the density-based noise application spatial clustering (DBSCAN) algorithm. The experimental results show that the real-time superpixel algorithm (50 fps) of DBSCAN clustering used by Shen is superior to the most advanced superpixel segmentation method in terms of accuracy and efficiency [5]. Ventura describes the method developed for creating computer-assisted dance choreography and explains how to apply choreography techniques to link the form and aesthetic aspects of movement, music, and video [6]. Chen incorporated the concept of deep learning into hyperspectral analysis for the first time. For the first time, a typical classification method based on hyperspectral information was used to verify the qualification of stacked autoencoders, and a new way of analyzing spatially dominant signals was again given. The joint hyperspectral spatial deep neural network it provides opens a new window for future in-depth research and shows the important potential of using spatial deep learning methods in accurate hyperspectral data classification [7]. Oshea introduced and discussed several new applications of physical layer deep learning (DL). By interpreting the communication system as an autoencoder, we have developed a basic new method that treats the communication system design as an end-to-end reconstruction task, aiming to jointly optimize the transmitter and receiver components in a single process [8]. Chung proposed an efficient local clustering algorithm that finds cuts by performing a scan over the hotkernel pagerank vector using the hotkernel pagerank approximation algorithm as a subroutine [9]. Yin designed an intrusion detection mechanism combining data stream clustering algorithm and an intrusion detection system to solve the problem of processing a large number of high-speed data streams. Through the clustering algorithm based on density and sliding window, the performance of processing data stream is improved, and the experiment shows that the efficiency of intrusion detection is higher than that of DenStream algorithm [10]. However, these scholars did not combine in-depth learning with the automatic arrangement of sports dance but only discussed its significance unilaterally.

The innovations of this article are mainly reflected as follows: (1) the development process of deep learning is introduced. As the latest research direction developed from artificial neural network technology in machine learning, deep learning has attracted widespread attention from the society. And we proposed a shallow regression model based on deep learning, given the general process of deep learning; (2) research on deep learning based on clustering algorithm and analysis of sports dance movement arrangement based on deep learning.

## 2. Method of Automatic Arrangement of Sports Dance Based on Deep Learning

### 2.1. Deep Learning

**Figure 1:**
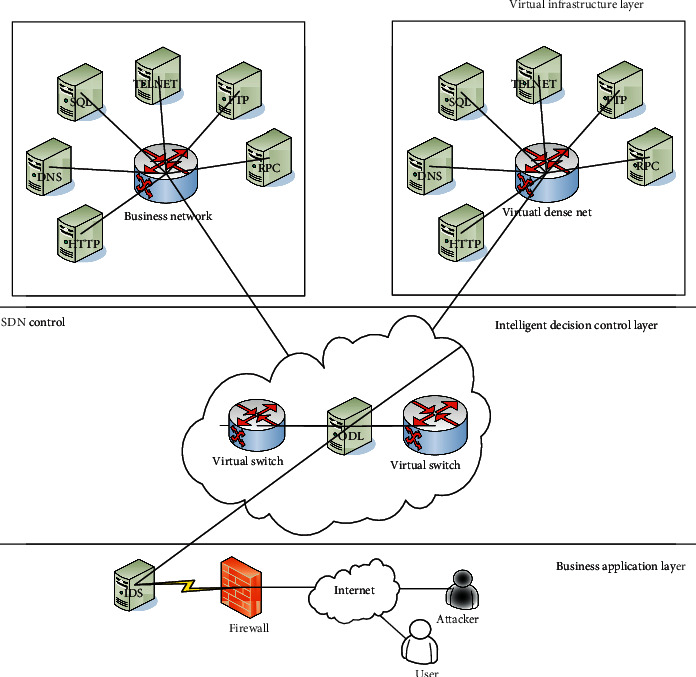
Virtual network optimization based on deep learning.

Deep learning is a new research direction in the field of machine learning. It has been introduced into machine learning to bring it closer to the original goal artificial intelligence. With the vigorous development of network information technology and massive news bursts, it has now entered the Internet information age. How to manage artificial intelligence scientifically, rationally, and effectively, and make it bring good services, has become an important field of competition in many service industries in modern society. In such a big environment, key technologies in the field of machine learning have attracted increasingly attention.  Figure 1 shows the virtual network optimization technology based on deep learning. At the same time, machine learning has also become a new direction, in which artificial neural network science and technology have flourished in mechanical teaching and have attracted widespread attention from the society [11].

**Figure 2:**
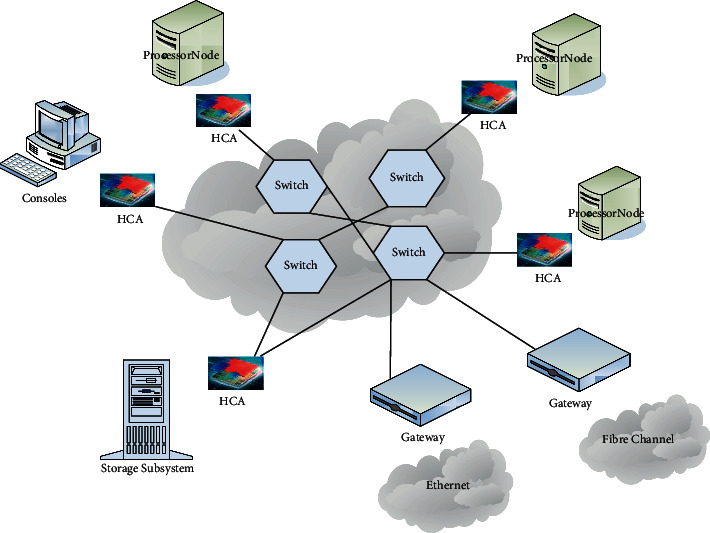
The field of deep learning.

The original neural network idea originated from the MCP artificial neuron modeling in 1943. The model simplified the neuron into three steps: input signal linearly weighted input; intermediate summation; nonlinear activation. The first calculation to study the application of MCP in the field of mechanical learning was in the perceptron calculation in 1958 [12]. The algorithm uses the MCP model, conducts a two-analysis of the orthogonality protection data provided by the training, and uses the gradient descent method to automatically learn information from the training samples and modify the connection weights [13]. In 1962, the method was proved to be theoretically convergent and thus promoted the first stage of scientific research on neural layer networks [14]. In 1989, in the handwritten character set recognition research, a backpropagation algorithm composed of a new cognitive machine, weight sharing, and convolutional neural layer was introduced, as well as the MP algorithm. Since then, this algorithm has also become the cornerstone of many deep learning models, which is also a major milestone in the development of convolutional neural networks [15]. After that, despite the long-term dormancy, neural networks, especially deep learning technology, have been bursting out in recent years [16]. Among them, in 2016, Alpha Go, developed by the Deep Mind research group of Google, went through five rounds of battles with world-famous chess masters and finally won the championship with a 4 : 1 result, bringing the social attention of deep learning technology to its peak [17].  Figure 2 shows the field based on deep learning. In China, new technology companies were represented by companies such as Baidu's Deep Learning Institute (IDL), and after the machine learning and artificial intelligence research and development teams of domestic universities have conducted in-depth research on deep learning technology, they have also produced a number of new technologies and new products that are widely valued [18]. Among them, deep learning is also widely used in other fields such as computer vision, speech recognition, and natural language processing.

### 2.2. Shallow Regression Model Based on Deep Learning

Deep learning is different from traditional shallow learning. The difference of deep learning is that it emphasizes the depth of the model structure. There are usually 5 layers, 6 layers, or even 10 layers of hidden nodes, which clarifies the importance of feature learning. Deep learning is a general term for a type of pattern analysis method. In terms of specific research content, it mainly involves three types of methods: neural network systems based on convolution operations, that is, convolutional neural networks, self-encoding neural networks based on multilayer neurons, and pretraining that is carried out in a multilayer self-encoding neural network and then combined with the identification information to further optimize the deep belief network of the neural network weight. Among the typical deep learning models are convolutional neural networks, DBN, and stack auto-encoding network models. The training process of deep learning is specifically divided into two steps: first, a single layer of neurons is constructed layer by layer, so that a single layer network is trained each time; when all layers are trained, the wake-sleep algorithm is used for tuning.

The method of regression analysis technology refers to the process of constructing a complex function between the independent variable and the dependent variable based on a large amount of training data and the use of mathematical means to represent the correlation mapping process. In the general return method, according to the difference of the function expression relationship between the independent variable and the dependent variable, the general return method can be divided into two categories: linear regression and nonlinear regression [19].

Among them, the basic model of unary linear regression is

(1) $$\begin{array}{c} g=\varphi_{1}a_{1}+\alpha . \end{array}$$

At the same time, *α* is the error, which can be regarded as the direct influence of other random factors; *φ*_1_ is the uncertainty parameter, which is the regression coefficient. We can know that the dependent variable *g* includes two parts: one is *φ*_1_*a*_1_, the part that changes the linear function of *g* caused by the change of a; the other is *α*. The main disadvantage of unary linear regression is that the return mode formed by image feature information cannot fully express the feature information of all images, and it has great limitations [20]. Therefore, multiple linear regression models are often used in most studies.

The multiple linear regression model refers to the linear relationship between several different independent variables and a dependent variable. The model can be expressed as

(2) $$\begin{array}{c} g=\varphi_{1}a_{1}+\varphi_{2}a_{2}+\cdots +\varphi_{m}a_{a}+\alpha ,\\ G\left(a,L_{h},\varphi^{h}\right)k_{n}\left(a^{h}\right)=0,\;n=1,2,\ldots ,N. \end{array}$$

The matrix description method of the multiple linear regression model is

(3) $$\begin{array}{c} G=A\varphi +\alpha . \end{array}$$

The classification surface can be described by the following equation:

(4) $$\begin{array}{c} g\left(a\right)=\mu^{E}a+c. \end{array}$$

At the same time, *μ* is the normal vector, which determines the orientation of the classification surface; *c* is the movement item, which determines the distance between the classification surface and the origin; *a* is the *m*-dimensional feature vector of the sample. When *g*(*a*)=0, the straight line can be regarded as the optimal classification surface [21].

(5) $$\begin{array}{c} \left\{\begin{array}{c} \mu^{E}a+c>0,\;y_{n}=+1,\\ \mu^{E}a+c<0,\;y_{n}=-1. \end{array}\right. \end{array}$$

When we have a new sample data for division, we can introduce it into the differential equation *μ*^*E*^*a*+*c*. If the result is assumed to be equal to zero, then it will be classified into type 1; otherwise, it will be type −1.

Among them, the support vector regression problem can be formalized as

(6) $$\begin{array}{c} \min\frac{1}{2}\mu^{2}+F\overset{k}{\underset{n=1}{\sum }}\left(\tau_{n}+\hat{\tau_{n}}\right),\\ \text{s}.t\;\left\{\begin{array}{c} g\left(a_{n}\right)-y_{n}\leq \alpha +\tau_{n}\\ y_{n}-g\left(a_{n}\right)\leq \alpha +\hat{\tau_{n}}\\ \tau_{n}\geq 0,\hat{\tau_{n}}\geq 0,\;n=\left(1,2,\ldots \right) \end{array}\right.,\\ g\left(a\right)=\overset{k}{\underset{n=1}{\sum }}\left(\hat{\beta_{n}}-\beta_{n}\right)h\left(a,a_{n}\right)+c,\\ \min G\left(a,\varphi^{h},L_{h}\right)=G\left(a,\varphi^{h},L_{h}\right). \end{array}$$

Among them, *h*(*a*_*n*_, *a*_*s*_)=∅(*a*_*n*_)^*E*^(*a*_*s*_) is called the kernel function.

In the research process of this article, the kernel function introduces the concept of Gaussian kernel function, and its expression is as follows:

(7) $$\begin{array}{c} h\left(a_{n},a_{s}\right)=\exp\left(-\frac{{\left\|a_{n}-a_{s}\right\|}^{2}}{2\partial^{2}}\right). \end{array}$$

Among them, ∂>0 is the width of the Gaussian kernel.

### 2.3. Convolutional Neural Networks and Offline Ranked Regression Models

**Figure 3:**
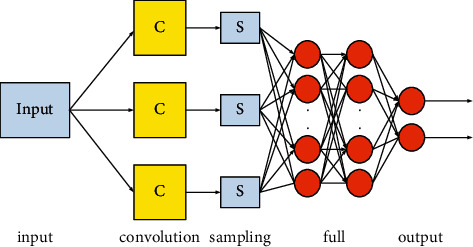
Basic structure diagram of convolutional neural network.

Machine learning is a multidomain interdisciplinary subject that studies how computers simulate or realize human learning behaviors to acquire new knowledge or skills, and to reorganize existing knowledge structures to continuously improve their performance. A deep learning framework, also known as a deep neural network, is a complex pattern recognition system that can perform functions ranging from automatic language translation to image recognition. Convolutional neural network (CNN) is a multilayer neural network.  Figure 3 shows the basic architecture of the convolutional neural network [22]. Among them, the application of convolutional neural networks is quite extensive, and it is widely used in image recognition, object recognition, behavior cognition, pose estimation, and neural style transfer in computer vision. Convolutional neural network is a type of feedforward neural network that includes convolution calculations and has a deep structure. It is one of the representative algorithms of deep learning. As a representative algorithm of deep learning, convolutional neural networks have the ability to characterize learning, as well as connectivity and biological similarity.

As shown in Figure 3, it can be seen that the convolutional neural network architecture is generally formed by five architectures: input and output layer, convolutional layer, sampling layer, fully connected layer, and input and output layer [23]. The functions of each level are described in detail as follows:

1. Input layer: the original data enters the convolutional neural network through the input layer. Generally speaking, when the convolutional neural network acts on image processing, the pixel value of the image can be used as direct input data [24].
2. Convolutional layer: also called feature extraction layer. In the classic CNN network structure, the convolution operation is generally a classic two-dimensional convolution operation, and each convolution operation of the input image will bring about a change in the spatial size. This layer can be used for deep extraction of the shallow features of the image [25].
3. Sampling layer: the main function is to eliminate the irrelevant and small interference that may be caused by each convolution operation, and to minimize the irrelevant data and its processing volume on the basis of retaining useful information, to achieve the purpose of accelerating the training network speed. The sampling process is performed independently in each depth dimension, so the depth of the image can be maintained constant. Generally speaking, commonly used sampling operations include maximum sampling and average sampling [26].
4. Full link layer: if you want to generate the final output, you must use the full link layer to get an output consistent with the number of target categories. There is a loss function similar to Soft max in the output layer, which can be used to estimate the prediction deviation. And if the forward pass is completed, the reverse pass layer will start to change the weight and bias value to reduce the bias and loss.
5. Output layer: after multilayer convolution and sampling, it is necessary to perform different operations on the image according to different purposes to determine the output form.

Convolution is a mathematical operation. Generally, the convolution between *g* and *y* can be expressed in the form of (*g∗y*)(*m*). Convolution can be divided into continuous and discrete cases. Their definition forms are as follows.

Continuous definition:

(8) $$\begin{array}{c} \left(g*y\right)\left(m\right)=\int_{-\infty }^{+\infty }g\left(a\right)y\left(m-a\right)\text{d}a. \end{array}$$

Discrete definition:

(9) $$\begin{array}{c} \left(g*y\right)\left(m\right)=\int_{a=-\infty }^{+\infty }g\left(a\right)y\left(m-a\right). \end{array}$$

The convolution operation of the convolutional neural network belongs to the discrete convolution, and the size and number of its convolution kernel can be flexibly defined.

Among them, the offline similarity ranking regression method is implemented based on the NDCG ranking model. This model is a classical model and is often used as an indicator to measure the quality of ranking. The commonly used formula is as follows:

(10) $$\begin{array}{c} \text{NDCG}=N_{M}^{-1}\times \text{DCG}=N_{M}^{-1}\times \underset{a\in \beta }{\sum }\frac{2^{t\left(a\right)}-1}{\log_{2}\left(1+\pi \left(a\right)\right)},\\ \beta G\left(a^{h},\varphi^{h},L^{h}\right)=0,\;m=1,2,\ldots ,s,\\ \varphi_{n}^{h+1}=\varphi_{n}^{h}+L_{h}k_{n}\left(a_{h}\right),\;n=1,2,\ldots ,N. \end{array}$$

Among them, *β* represents the sample block patch in the video frame; *t*(*a*) represents the area coverage ratio between the current sample block and the manually selected standard block; *π*(*a*) represents the position of the current sample block in the overall ranking; *N*_*M*_ is the largest normalization item in DCG. When the area coverage ratios of all sample blocks and standard blocks are arranged in descending order, the maximum value of DCG/NDCG can be obtained, and the value range of NDCG is [0,1]. Among them, to fully introduce the offline ranking learning regression method of the entire similarity function, we will elaborate from two aspects: the traditional stochastic gradient algorithm and the approximate gradient learning algorithm.

First, the traditional stochastic gradient descent (SGD) method is introduced. The general optimization process cannot be directly used to obtain the gradient, and it needs to be reexpressed by approximation.

(11) $$\begin{array}{c} \pi \left(a\right)\approx 1+\underset{b\neq a,b\in \beta }{\sum }\text{sign}\left(g\left(\hat{a},\hat{b}\right)\right)=1+\underset{b\neq a,b\in \beta }{\sum }\text{sign}\left(g\left(\hat{b}\right)-\left(\hat{a}\right)\right),\\ \max\left|k_{n}\left(a^{h}\right)\right|<\sigma ,\;1<n<N. \end{array}$$

Among them, $\hat{a}$ represents the potential feature vector obtained by the current sample block patch from the CNN model, and $g\left(\hat{a}\right)$ represents the similarity of the current sample block patch. $\text{sign}\left(g\left(\hat{b}\right)-\left(\hat{a}\right)\right)$ represents the signum function. When $g\left(\hat{b}\right)$ is not less than $g\left(\hat{a}\right)$ , it is a positive value; otherwise, it is a negative value.

**Figure 4:**
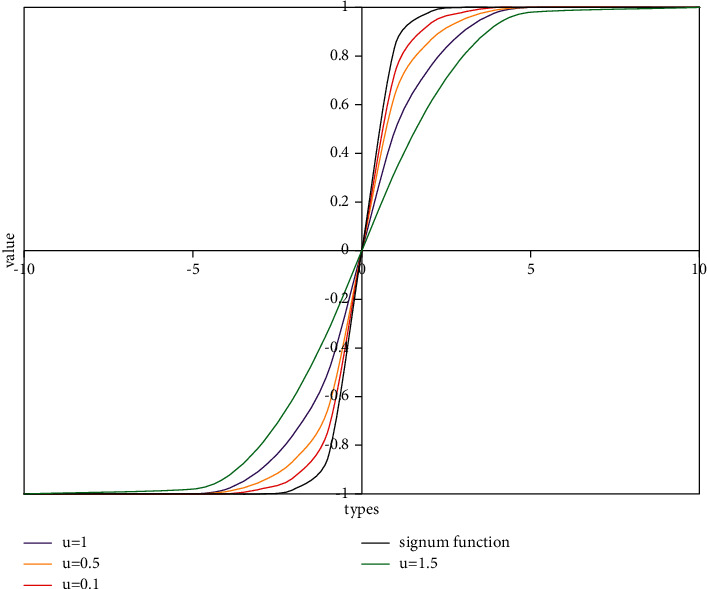
Curve change of signum function when *u* value is different.

In addition, because the signum function is not 1 or −1 step characteristic, the function can be further improved by processing like $\text{sign}\left(\varphi \right)\approx \varphi /\sqrt{\varphi^{2}+\mu^{2}}$. Among them, *φ* represents the function variable, which controls the smoothness of the improved function. It can be easily seen from Figure 4 that the smaller the *μ* is, the closer the improved function is to the original signum function. In this way, an improved position function can be obtained.

(12) $$\begin{array}{c} \pi \left(a\right)\approx 1+\underset{b\neq a,b\in \beta }{\sum }\text{sign}\left(g\left(\hat{a},\hat{b}\right)\right)=1+\underset{b\neq a,b\in \beta }{\sum }\frac{g\left(\hat{a},\hat{b}\right)}{\sqrt{g^{2}\left(\hat{a},\hat{b}\right)+\mu^{2}}}. \end{array}$$

At the same time, a new model can be derived:

(13) $$\begin{array}{c} \text{eNDCG}\left(a\right)=N_{M}^{-1}\times \sum_{a\in \beta }\frac{2^{t\left(a\right)}-1}{\log_{2}\left(2+\sum_{b\neq a,b\in \beta }g\left(\hat{a},\hat{b}\right)/\sqrt{g^{2}\left(\hat{a},\hat{b}\right)+\mu^{2}}\right)}. \end{array}$$

On the basis of the above formula, by using the chain rule twice in succession, a new gradient function expression can be obtained:

(14) $$\begin{array}{c} \frac{\text{eNDCG}\left(a\right)}{\partial \varepsilon }=N_{M}^{-1}\underset{a\in \beta }{\sum }\left(-\frac{2^{t\left(a\right)}-1}{\log_{2}^{2}\left(1+\pi \left(a\right)\right)}\cdot \frac{1}{\left(1+\pi \left(a\right)In2\right)}\right)\cdot \left(\underset{b\neq a,b\in \beta }{\sum }\frac{\mu^{2}}{{\left(\sqrt{g^{2}\left(\hat{a},\hat{b}\right)+\mu^{2}}\right)}^{3/2}}\cdot \frac{\partial g\left(\hat{a},\hat{b}\right)}{\partial \varepsilon }\right). \end{array}$$

Thus, based on the gradient expression shown in the above formula, end-to-end learning of the model based on the SGD algorithm can be realized.

Among them, the more effective near-end gradient descent (PGD) algorithm can be used to realize the sorting regression learning of the offline similarity function. Its central idea mainly uses the regularization problem with the form of LASSO to solve the similarity learning problem. The problem can be expressed by the following formula:

(15) $$\begin{array}{c} \min{\left(B-N_{M}^{-1}\times \sum_{a\in \beta }\frac{2^{t\left(a\right)}-1}{\log_{2}\left(2+\sum_{b\neq a,b\in \beta }g\left(\hat{a},\hat{b}\right)/\sqrt{g^{2}\left(\hat{a},\hat{b}\right)+\mu^{2}}\right)}\right)}^{2}+\beta \varphi =\min G\left(\varphi \right)+\beta \varphi , \end{array}$$

In the formula, B is the ideal NDCG value in the sample block patch sorting list; *β* is the regularized control coefficient; *G*(*φ*) is the loss function.

To quickly solve the above-mentioned LASSO problem, the near-end gradient descent algorithm (PGD) can be used. The PGD algorithm is also an iterative algorithm: in each of its individual iterations, after obtaining the learning result *φ*_*h*−1_ from the *h* − 1 th iteration, it is immediately sent to the following formula to solve the intermediate variable *S*_*h*−1_.

(16) $$\begin{array}{c} S_{h-1}=\varphi_{h-1}-\frac{1}{T}\nabla G\left(\varphi_{h-1}\right). \end{array}$$

Among them, *T* represents the positive scale factor, and ∇ represents the gradient. Here, the idea of soft threshold can be used to calculate the nth dimension of the *h*th iteration *φ* in the formula, as shown in the following formula:

(17) $$\begin{array}{c} \varphi_{h}^{n}=s_{h-1}^{n}-\frac{\beta }{T}\cdot \max\left\{\left|s_{h-1}^{n}\right|-\frac{\beta }{T},0\right\}. \end{array}$$

Among them, max represents the maximum function; *s*_*h*−1_^*n*^ represents the value of the nth dimension of *S*_*h*−1_ obtained from the above formula. In addition, the formula can also be extended in the form of the following piecewise function:

(18) $$\begin{array}{c} \left\{\begin{array}{cc} s_{h-1}^{n}-\frac{\beta }{T}, & \frac{\beta }{T}<s_{h-1}^{n},\\ 0, & \left|s_{h-1}^{n}\right|\leq \frac{\beta }{T},\\ s_{h-1}^{n}+\frac{\beta }{T}, & s_{h-1}^{n}<-\frac{\beta }{T}. \end{array}\right. \end{array}$$

Through the above method, the nth dimension of *φ* in the *h*th iteration can be quickly calculated. When the stop condition of *φ*_*i*_ − *φ*_*i*−1max_ ≤ to is met, the iteration in the gradient descent will be terminated.

**Figure 5:**
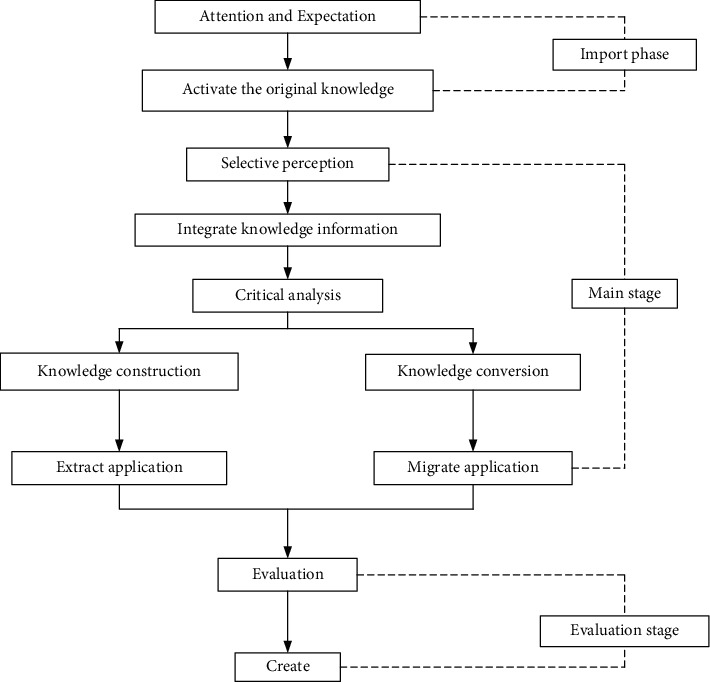
General process model of deep learning.

In summary, the offline similarity ranking regression learned in this section refers to the classic NDCG ranking model, which is discrete and nondifferentiable and is not suitable for the optimal solution. Therefore, this paper proposes a continuous and differentiable ranking model called eNDCG. In addition, by deriving the explicit expression of the gradient in the stochastic gradient algorithm and proposing an approximate gradient descent algorithm based on the LASSO regularization optimization problem, the self-learning of the similarity function in the end-to-end learning process of the model is realized by both traditional and fast solution methods. It is worth emphasizing that these two algorithms can be implemented in deep learning back propagation, but the latter is more efficient.  Figure 5 shows the general process model of deep learning.

## 3. Experimental Results of Research on Automatic Arrangement of Sports Dance Movements Based on Deep Learning

### 3.1. Deep Learning Based on Clustering Algorithm

To explore the effect of deep learning in the research of automatic choreography of sports dance, the analysis and research of deep learning training based on clustering algorithm are firstly carried out. The following are the analysis and research of its training results. Cluster analysis, also known as group analysis, is a statistical analysis method for studying (sample or index) classification problems and is also an important algorithm for data mining.

**Figure 6:**
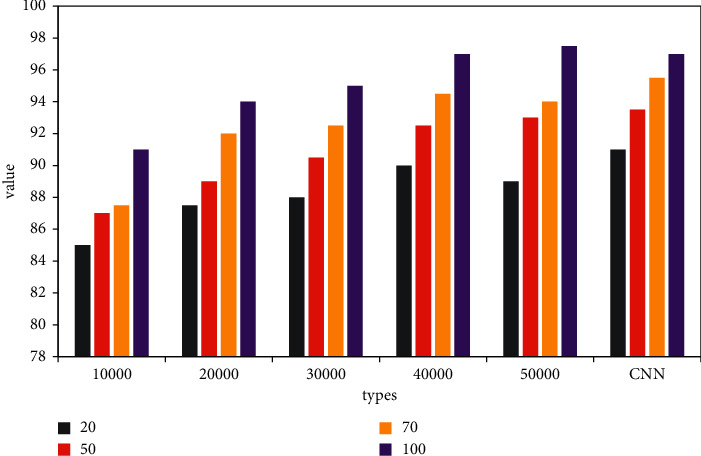
Comparison of *k*-means improvement and CNN accuracy.

In this paper, the improvement of deep learning training based on the clustering algorithm is indeed effective for fast training with a small number of samples. As a module in data mining, the clustering algorithm can be used as a separate tool to discover some deep information distributed in the database. And we summarize the characteristics of each category or focus on a specific category for further analysis. Moreover, cluster analysis can also be used as a preprocessing step for other analysis algorithms in data mining algorithms. Cluster analysis refers to the analysis process of grouping a collection of physical or abstract objects into multiple classes composed of similar objects. It is an important human behavior. The goal of cluster analysis is to collect data to classify on the basis of similarity. For the *k*-means clustering method, as shown in Figure 6, when the clustering samples are more than 30,000, the result of the deep learning network improved by the *k*-means clustering algorithm is only slightly lower than the original CNN network. However, the average time of *k*-means improved network is about 1 hour less than the original CNN. Analyze *k*-means to improve the network. As the number of network samples increases, and the hidden layer of the network increases, the accuracy of network testing is gradually improving. However, the time consumption rate is gradually increasing. After the clustering sample exceeds 30,000, the test accuracy of the network is almost unchanged, but when the sample is 50,000, it takes about 1 hour more on average than the sample of 30,000.

It can be seen from the GMM clustering method that when the number of clustering samples is the same as the original CNN training samples, that is, 50000, the accuracy of the GMM improved network is higher than that of the original CNN network. However, the time taken by the GMM algorithm is lower than the time taken by the original CNN network training. However, due to the randomness of GMM in the clustering process, the accuracy of GMM's improved network is constantly fluctuating. Analyze GMM to improve the network. As the number of network samples increases, and the hidden layer of the network increases, the accuracy of network testing is gradually increasing, and the time consumption rate is gradually increasing. On average, for every additional 10,000 samples, it takes 0.2 hours more time.

**Figure 7:**
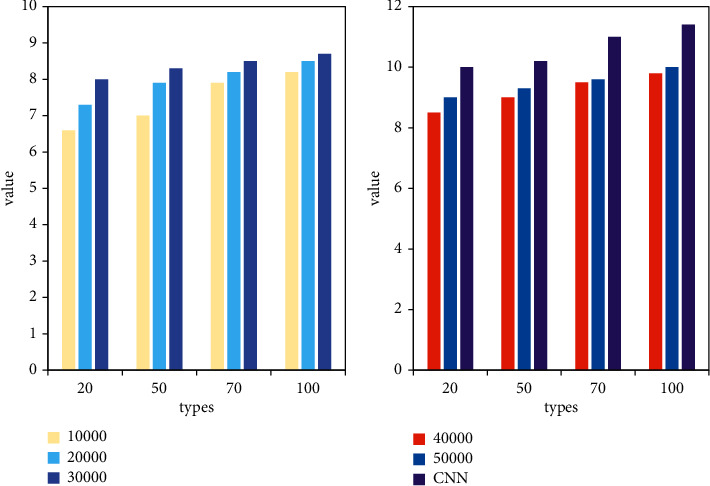
Comparison of *k*-means improvement and CNN time.

For the *K*-nearest neighbor clustering method, as shown in Figure 7, when the clustering samples are more than 40,000, the result of the deep learning network improved by the *K*-nearest neighbor clustering algorithm is only slightly lower than the original CNN network. However, the average time of the improved *K*-nearest neighbor network is about 1 hour less than that of the original CNN. Analyze *K*-nearest neighbors to improve the network. As the number of network samples increases, and the hidden layer of the network increases, the accuracy of network testing is gradually improving. However, the time consumption rate is gradually increasing. After the clustering sample exceeds 40,000, the test accuracy of the network is almost unchanged, but when the sample is 50,000, it takes about 0.4 hours more than the sample of 40,000 on average.

**Figure 8:**
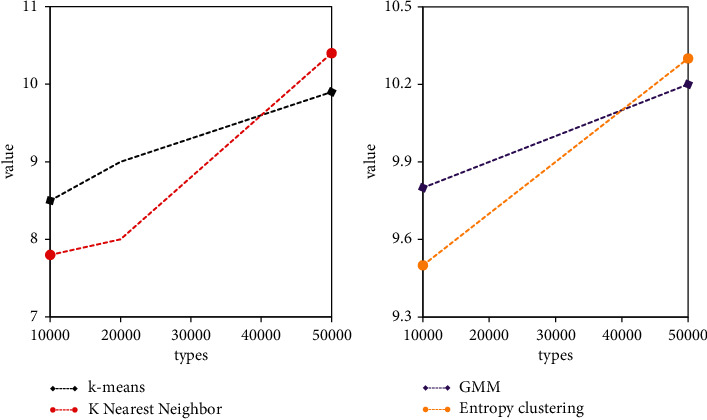
Improved algorithm time comparison.

In the entropy clustering method, as shown in Figure 8, when the clustering samples are more than 40,000, the result of the deep learning network improved by the entropy clustering algorithm is slightly higher than that of the original CNN network. However, the average time of the entropy clustering improved network is about 0.4 hours less than the original CNN. Analyze entropy clustering to improve the network. As the number of network samples increases, and the hidden layer of the network increases, the accuracy of network testing is gradually increasing, but the time-consuming rate is also gradually increasing. When the clustering sample exceeds 40,000, the test accuracy of the network is almost unchanged, but when the sample is 50,000, it takes about 0.3 hours more than the sample of 40,000 on average.

Analyzing the improved network of four clustering algorithms, taking 100 hidden layers as an example, in terms of time utilization rate, *K*-nearest neighbors are the lowest, and GMM has the highest time utilization rate due to the complexity of calculation. However, when the number of samples is 50000, *k*-means clustering is the lowest, and entropy clustering is the highest. Analyzing the reasons, it is found that the computational complexity of entropy clustering increases as the number of samples increases. In terms of accuracy, entropy clustering is the highest overall, However, when the number of samples is 30,000, *k*-means clustering is the highest. When the number of samples reaches about 50,000, the accuracy of the four improved networks is almost the same.

**Figure 9:**
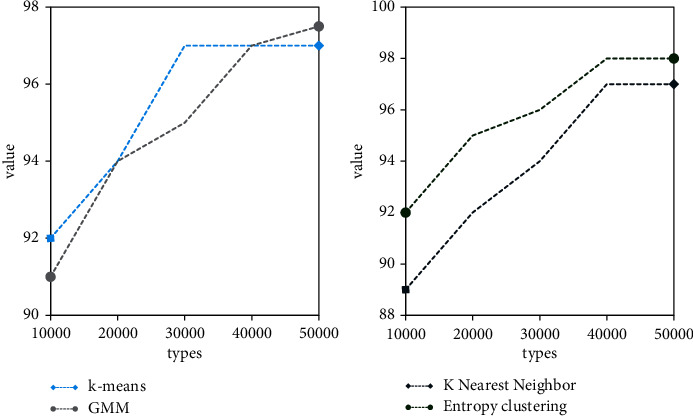
Comparison of improved algorithm accuracy.

As shown in Figure 9, when the number of samples is 30,000, the difference between the accuracy rate and the original training result is about 2%, but the training time is shortened by about 2 hours on average. Experiments prove that the improvement of deep learning training based on the clustering algorithm proposed in this paper improves the training speed and saves training time on the basis of ensuring the accuracy of training with a small number of samples.

When the number of samples is 40,000, the accuracy is the same as the original training result, but the training time is shortened by about 1 hour on average. When the number of samples is 30,000, the difference between the accuracy rate and the original training result is about 2%, but the training time is shortened by about 2 hours on average. Among the four improved algorithms, the accuracy of the *k*-means algorithm will not increase significantly as the number of samples increases after the data volume is 30,000. However, after the data volume of other methods is 40,000, the accuracy rate will not increase significantly with the increase of the number of samples. When the amount of data is 30,000, the accuracy of the improved method of *k*-means clustering algorithm is higher than that of the other three. This is because the DDSM sample library is mostly benign mass, and the edges of benign mass are clear, which is suitable for the *k*-means clustering algorithm in terms of images. From the analysis in the figure, it can be seen that the improved network of entropy clustering algorithm proposed in this paper is significantly better than the other three clustering algorithms in terms of accuracy.

The basic idea of k-medoid algorithm is to first find a representative object for each cluster to determine *k* clusters. If replacing a cluster representative can improve the quality of the obtained partition, then the old representative object can be replaced with a new representative object. The processing process of the *k*-means algorithm is as follows: first, *k* objects are randomly selected as the centroids of the initial *k* clusters. The remaining objects are then assigned to the nearest clusters according to their distances from the centroids of the respective clusters; finally, the centroids of the respective clusters are recalculated. Repeat this process until the objective function is the smallest. The improved network of entropy clustering algorithm proposed in this paper has more advantages.

### 3.2. Sports Dance Movement Choreography

Through in-depth exercises, this chapter analyzes the physical dynamics of the complete set of sports art dance dynamics, including the physical dynamics of the complete set of dynamic main design styles and the second design style dance activities. And when playing video data, the whole set of dancing dynamics is divided into operational dynamics, lifting gestures, conversion and connection dynamics, second-style street dance movements, etc. The number of beats and the completion time of each segment of the operation after the split are counted, and the data of each segment is added to obtain the total number of beats and the time of operation, which are used for the comparative analysis of this article.

It can be seen from Table 1 that, in terms of the number of actions, the total number of beats of the complete set of actions of Team D is 220 beats, which is the largest number of complete sets of actions of all teams. The total number of beats of its manipulation action is 118, accounting for 54.38% of the total ranking fourth in the art score; the total number of beats of the complete set of actions of Team A is 201 beats, which is the least number of complete sets of all teams. The total number of beats of the complete set of actions of team A is 109 beats, accounting for 56.68% of the total beats of the set of actions, ranking first in the art score; the total number of beats of the complete set of actions of Team B is 202, which ranks third in the total number. The total number of beats of exercise is 106, which is the least number of exercises of all teams, accounting for 53.11% of the total, ranking second in art scores; Team C has a total of 211 beats, ranking second in total number, and 131 beats in total exercises, which is the largest number of exercises among all teams, accounting for 59.79% of the total, ranking third in art scores.

It can be seen from Table 2 that the total time for the complete set of actions of the four participating teams is within the range of 81 to 83 seconds, which meets the time requirements in the competition rules. From the point of view of the time spent on the exercises, the A team's exercises took 44 seconds, accounting for 50.2% of the total time spent on the set of exercises; the B team's exercises took 40 seconds, accounting for 50% of the total time spent on the set of exercises; Team C took 59 seconds, accounting for 74.01% of the total time of the complete set of exercises; Team D took 47 seconds, accounting for 55.68% of the total time of the complete set of movements.

From the comparison in Table 3, it can be seen that, among the continuous operation units displayed by Team A, 2 operation units are continuously displayed the most, showing multiple groups of high-intensity continuous operation units. And in the complete set of exercises, the intensity distribution of the exercise is stabilized, so that the complete set of exercises maintains an appropriate intensity, and the complexity and continuity of the complete set of exercises are ensured.

It can be seen from Table 4 that the number of street dance moves of team C is 34 shots, which took 14 seconds, which is the team with the shortest time among the participating teams. The number of street dance moves of Team A is 50 shots, and it takes 24 seconds, which is the team with the longest time for the second style dance moves; from the perspective of the proportion of the number of street dance moves in the set of moves, the number of street dance moves of Team A accounted for 26.49% of the set of moves, which is the highest proportion of street dance moves among the four teams. Secondly, Team D occupies 21.36%, again Team B 19.19%, and the least is Team C 16.35%; Team A and Team D reach 6 operational units, followed by 5 operational units of Team B. Team C has only 4 exercise units. The more the exercise units, the greater the intensity of dance movements. Compared with the other three teams, the intensity of dance movements of team C is smaller.

**Figure 10:**
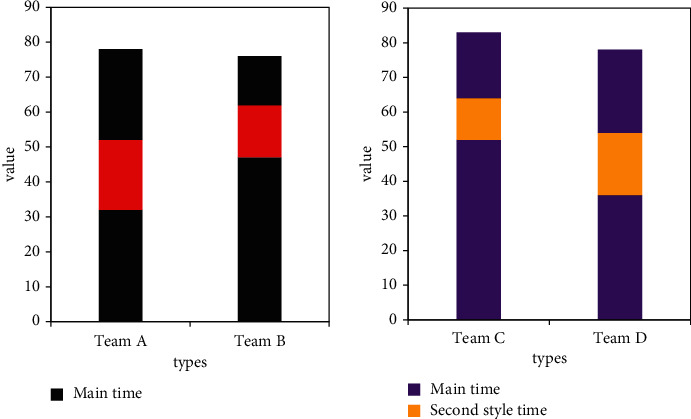
Distribution of the second style dance moves.

At the same time, it can be seen from Figure 10 that, in terms of the timing of the start of the second style dance movement, Teams A and D chose to arrange the second style dance movement in the middle of the set of movements. Compared with Team D, Team A started earlier, while Teams B and C chose to insert the second-style dance moves in the latter part of the set of moves, and the second-style dance moves of Team C started later. Experimental research shows that deep learning can effectively improve the artistic innovation ability of automatic arrangement of sports dance movements and increase the accuracy of dance movements by 80%.

## 4. Discussion

According to the analysis of the research data, there must be at least 50% of the exercises in the complete set of exercises, but the proportion should not be too large. And we choose the display of 2 consecutive operation units as the main and should try to reduce more than 3 or 4 operation units in succession, so that the operation actions can be evenly distributed in the set. The rules stipulate that the number of lifting dynamics is at most one, so the arrangement of the lifting dynamics is particularly important. In the new cycle, the lifting dynamics will gradually change from pure dynamic modeling to derive from various types of lifting dynamics. Although there is no specific number of kinetic collocation actions in the complete set of actions, it should be used in strict accordance with the complete set of action times, and the innovative conversion and cohesive actions and dynamic collocation dynamics and other elements should be properly connected. At the same time, players and coaches should try more innovative forms of coordination in the complete set of arrangements to enhance the fullness of the overall style of the complete set, thereby further enhancing the artistic effect of the complete set.

## 5. Conclusions

The article mainly analyzes the characteristics of the current sports dance movement, finds out the essential attributes of the sports dance technology, and proposes the basic path to overcome the nonstandardized content of dance education and training and the irrational teaching methods. That is, we must first thoroughly explore the essential attributes of sports dance itself and then determine the effective auxiliary teaching methods that best meet the characteristics of the current sports dance. According to the comprehensive influence on the characteristics of the current sports dance, we do a reasonable allocation of the time ratio of effective teaching methods, to improve the level of the players in the most targeted way and jump out of the world's high-level sports dance. On the basis of deep learning, we strengthen practical ability on the basis of consolidating theory, further improve one's own business ability and educational technology level, and actively absorb advanced teaching methods. We earnestly study reasonable teaching methods and apply them to curriculum training practice, actively gaining insights into new development trends in educational methods and skills, to enhance the artistic creativity of students' arrangements. It is expected to provide theoretical support for cultivating the athletic ability of sports dancers and the development of sports dance careers, and to provide certain contributions to the research on automatic choreography of sports dance movements. Due to the incompleteness of the existing technology, there are still certain shortcomings in the research on the automatic arrangement of sports dance movements based on deep learning, and we will further carry out more in-depth research in the follow-up.

## Figures and Tables

**Table 1:** Statistics of the number of individual dance movements.

Ranking	Team	Total number of action beats	Total number of beats of the complete set of actions	Proportion of exercises (%)
1	A	109	201	56.68
2	B	106	202	53.11
3	C	131	211	59.79
4	D	118	220	54.38
	Average value	121	201	57.62

**Table 2:** Statistics of the time consumed by individual dance exercises.

Ranking	Team	Total time of complete set of actions (seconds)	Operational time (seconds)	Operating time percentage (%)	Beat rate
1	A	83	44	50.2	2.71
2	B	80	40	50	2.58
3	C	81	59	74.01	2.09
4	D	82	47	55.68	2.65

**Table 3:** Statistics of dance individual final exercises.

Ranking	Team	Number of operating units	Number of continuous operation units	2 continuous operation units		3 continuous operation units		More than 4 operation units	
				N (group)	%	N (group)	%	N (group)	%
1	A	15	8	7	86.02	1	14.35	—	—
2	B	12	5	3	60	1	20	1	20
3	C	17	6	2	33.3	2	33.3	2	33.3
4	D	15	6	4	66.6	1	16.6	1	16.6

**Table 4:** Statistics of the second style dance movements in the dance individual finals.

Ranking	Team	Timing of the start of the second style dance (seconds)	Second style dance movement time (seconds)	The second style dance movement beats (beats)		The number of the second style dance movement exercise unit (units)
				N	%	
1	A	29	24	50	26.49	6
2	B	51	18	39	19.19	5
3	C	49	14	34	16.35	4
4	D	38	21	49	21.36	6

## Data Availability

All the data used is given in the paper.
